# RWD-Cockpit: Application for Quality Assessment of Real-world Data

**DOI:** 10.2196/29920

**Published:** 2022-10-18

**Authors:** Lmar Marie Babrak, Erand Smakaj, Teyfik Agac, Petra Maria Asprion, Frank Grimberg, Daan Van der Werf, Erwin Willem van Ginkel, Deniz David Tosoni, Ieuan Clay, Markus Degen, Dominique Brodbeck, Eriberto Noel Natali, Erik Schkommodau, Enkelejda Miho

**Affiliations:** 1 University of Applied Sciences and Arts Northwestern Switzerland, School of Life Sciences Muttenz Switzerland; 2 Fachhochschule Nordwestschweiz University of Applied Sciences and Arts Northwestern Switzerland, School of Business Olten Switzerland; 3 Q-Strip Steenbergen Netherlands; 4 Evidation Health Inc San Mateo, CA United States; 5 aiNET GmbH Basel Switzerland; 6 Swiss Institute of Bioinformatics Lausanne Switzerland

**Keywords:** real-world data, real-world evidence, quality assessment, application, mobile phone

## Abstract

**Background:**

Digital technologies are transforming the health care system. A large part of information is generated as real-world data (RWD). Data from electronic health records and digital biomarkers have the potential to reveal associations between the benefits and adverse events of medicines, establish new patient-stratification principles, expose unknown disease correlations, and inform on preventive measures. The impact for health care payers and providers, the biopharmaceutical industry, and governments is massive in terms of health outcomes, quality of care, and cost. However, a framework to assess the preliminary quality of RWD is missing, thus hindering the conduct of population-based observational studies to support regulatory decision-making and real-world evidence.

**Objective:**

To address the need to qualify RWD, we aimed to build a web application as a tool to translate characterization of some quality parameters of RWD into a metric and propose a standard framework for evaluating the quality of the RWD.

**Methods:**

The RWD-Cockpit systematically scores data sets based on proposed quality metrics and customizable variables chosen by the user. Sleep RWD generated de novo and publicly available data sets were used to validate the usability and applicability of the web application. The RWD quality score is based on the evaluation of 7 variables: *manageability* specifies access and publication status; *complexity* defines univariate, multivariate, and longitudinal data; *sample size* indicates the size of the sample or samples; *privacy and liability* stipulates privacy rules; *accessibility* specifies how the data set can be accessed and to what granularity; *periodicity* specifies how often the data set is updated; and *standardization* specifies whether the data set adheres to any specific technical or metadata standard. These variables are associated with several descriptors that define specific characteristics of the data set.

**Results:**

To address the need to qualify RWD, we built the RWD-Cockpit web application, which proposes a framework and applies a common standard for a preliminary evaluation of RWD quality across data sets—molecular, phenotypical, and social—and proposes a standard that can be further personalized by the community retaining an internal standard. Applied to 2 different case studies—*de novo*–generated sleep data and publicly available data sets—the RWD-Cockpit could identify and provide researchers with variables that might increase quality.

**Conclusions:**

The results from the application of the framework of RWD metrics implemented in the RWD-Cockpit application suggests that multiple data sets can be preliminarily evaluated in terms of quality using the proposed metrics. The output scores—quality identifiers—provide a first quality assessment for the use of RWD. Although extensive challenges remain to be addressed to set RWD quality standards, our proposal can serve as an initial blueprint for community efforts in the characterization of RWD quality for regulated settings.

## Introduction

### Background

Real-world data (RWD) is defined as health care data generated outside of randomized controlled trials (RCTs) [1]. Real-world evidence (RWE) regarding the use, benefits, and risks of medications is obtained through comprehensive analyses and validation of RWD. Examples of RWD include electronic health records, prescription and billing data, insurance claims, genetic and molecular biobanks, medical-related products, disease registries, and patient-generated health data collected through a variety of sources and digital devices such as wearables and smartphones [2,3]. RWD emerged through the widespread use of health-related apps, implementation of electronic health records in hospitals, and routine genetic testing. Recently, these data were recognized as a valuable resource for biopharmaceutical companies to reduce research and development expenditures, and this has been primarily implemented by regulatory agencies in postmarket analysis of medical products [4].

RWE can supplement, and has often served as, primary data to inform on regulatory decisions such as alternative drug indications and is used in orphan and oncological disease studies [5]. In response to this trend, regulatory agencies such as the US Food and Drug Administration (FDA) and European Medicines Agency (EMA) have implemented strategies for the inclusion of RWD and RWE as part of their regulatory approach to digitalization in health care to inform regulatory decisions such as late-term adverse effects or stratifying clinical trial population groups with the US 21st Century Cures Act [6] and the EMA Regulatory Science to 2025 strategy [7]. Several studies have shown that the use of RWD in determining patient health status, especially in cases of progressive or chronic diseases such as Alzheimer disease and Parkinson disease, can greatly affect current diagnosis and prognosis as well as optimize disease management [3,8]. The use of RWE is also crucial for assessing the safety and effectiveness of processes that cannot be appropriately addressed in an RCT, such as surgical procedures [9]. Ongoing efforts by the regulatory agencies have already seen practical implementations of RWD used to receive regulatory approval as an alternative to RCTs. For example, Prograf (tacrolimus), a drug initially approved to prevent organ rejection in liver transplantations, has received FDA approval for use in kidney and heart transplantations [10] and similar approvals in Europe [11]. These cases and others reflect how well-designed studies relying on fit-for-purpose RWD can be considered adequate under FDA and EMA regulations [12]. To maximize the implementation of RWE, an important challenge currently is to find data that provide the most suitable measurements for biopharmaceutical companies and regulatory agencies [13].

### Objectives

**Figure 1:**
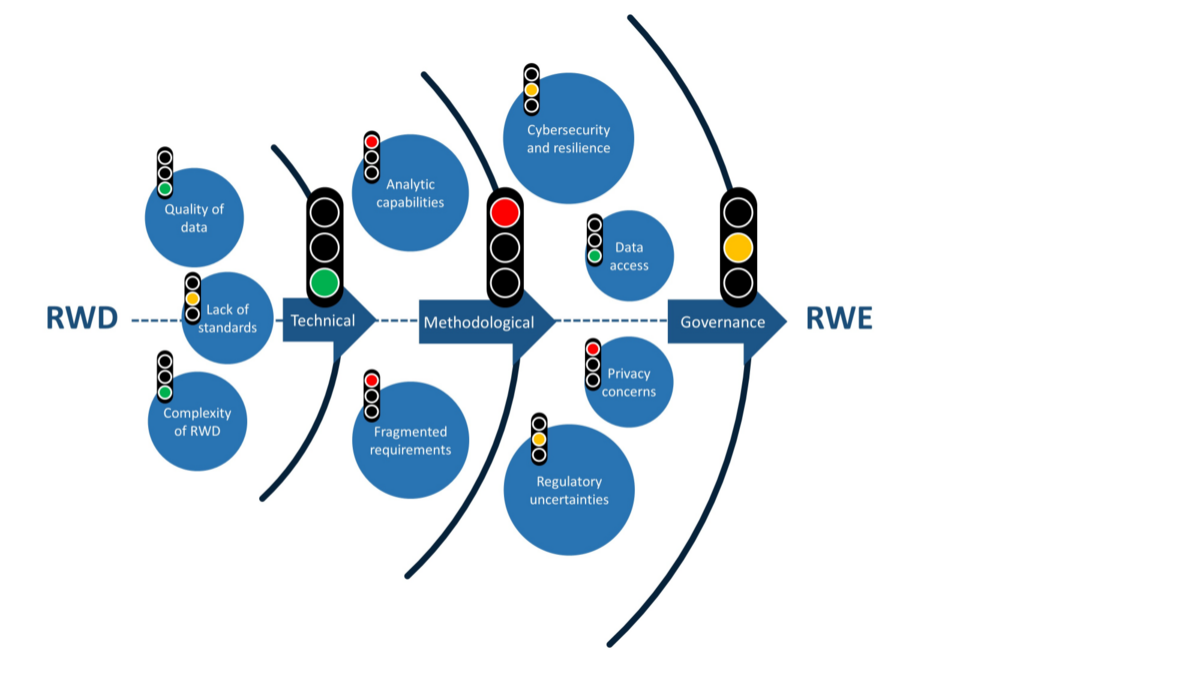
Challenges in the transformation of real-world data (RWD) to real-world evidence (RWE).

**Figure 2:**
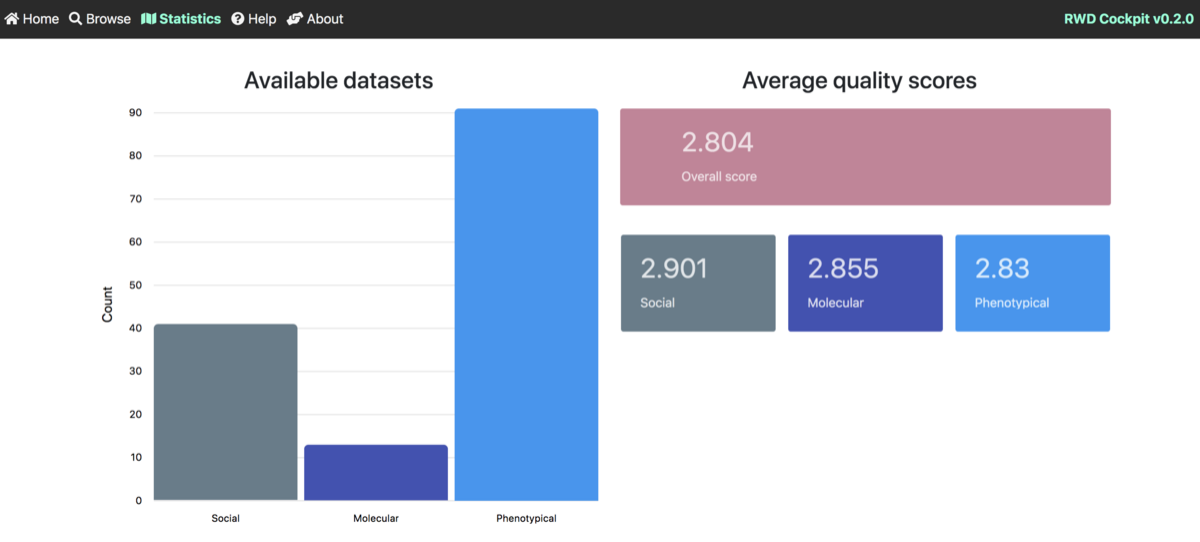
Screenshot of the Statistics tab of the RWD-Cockpit web-application of the overall score of RWD data sets. A data set tree map allocated under the general statistics bar chart enables the sequential selection of the type of data, its complexity, assessment and other parameters for the identification of the RWD and the quality needed.

**Figure 3:**
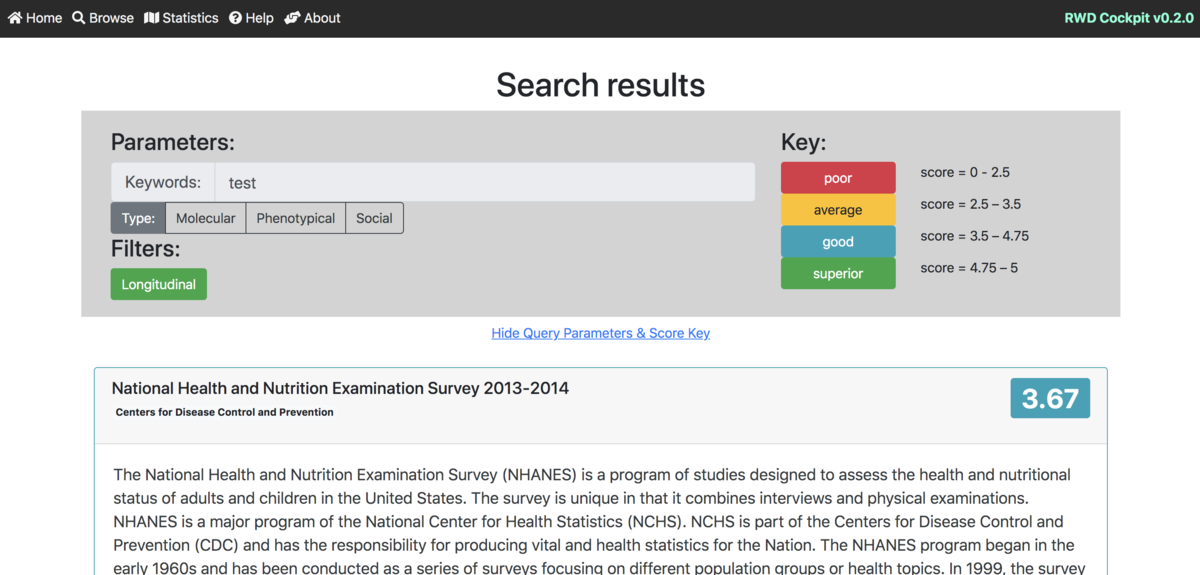
Search results with the keyword test in data sets filtered by the variable complexity and descriptor longitudinal. The first, highest-score data set entry is shown.

The sources and types of RWD are diverse, ranging from medication orders to patient-generated (eg, PatientsLikeMe and Carenity), digitally collected (fitness trackers), and social media data [14]. However, the criteria used by the biopharmaceutical industry to select appropriate data sets for different applications compared with traditional RCTs are unclear [12]. In addition, data origin, diversity, and complexity make it difficult to consistently rank and assess RWD quality [15]. Lack of standardization and structure among data sets augments and lengthens the process of identifying the right fit-for-purpose RWD and generating meaningful analyses [16]. Carefully curated, validated, standardized, and high-quality data are needed to generate widely accepted RWE that can bridge the knowledge gap between standardized RCTs and the real world. To date, there are neither clear standards nor available tools to assess RWD quality [17]. To address these challenges (Figure 1), we have created an easy-to-use, accessible web application tool that assesses RWD data sets using a customizable selection of proposed standard variables: the *RWD-Cockpit* (Figures 2 and 3).

## Methods

### Overview

A total of 106 RWD data sets were selected as a target sample group for the development of a scoring method in the RWD-Cockpit to assess RWD data sets. The metadata of these data sets and publications were investigated with regard to data quality to devise a scoring method to assess the quality of RWD. The scoring method takes into account seven variable metrics for data-quality assessment:

1. Manageability specifies the access rights that users may or may not have for a data set as well as whether the data have been published in a peer-reviewed journal.
2. Complexity defines whether the data set is univariate, multivariate, or longitudinal.
3. Sample size defines the sample size of a given data set.
4. Privacy and liability stipulates privacy rules according to the data context of use.
5. Accessibility specifies how the data set can be accessed and to what granularity.
6. Periodicity specifies how often the data set is updated.
7. Standardization specifies whether the data set adheres to any specific technical or metadata standard.

For each variable, there are several associated *descriptors* that define specific characteristics of the data set. The descriptors are explained in detail in Table 1. The RWD scoring formula subsequently averages the performance of variables for a given data set to assign a final score. A specific score (0 to 100) is assigned to each variable’s descriptor. Each variable can have >1 descriptor (eg, the data set contains longitudinal and multivariate data), and an average score for each variable is taken. In the *Complexity* variable, multivariate or univariate can be chosen. Subsequently, a cumulative average is calculated for all variables. This cumulative average is normalized to a score from 1 to 5, with 5 being the best quality for a data set. This score is called the quality identifier and is displayed and associated with each data set. The normalization is performed by dividing the cumulative score by 7 (number of descriptors), then dividing by 100 and multiplying by 5. An example of the scoring methodology for 2 data sets is shown in Table 2.

**Table 1:** Variable and descriptor definitions and real-world data examples.

Variable and descriptor (scored highest to lowest)		Definition	Examples
**Manageability: describes the level of data management, such as whether the data are protected; have been peer reviewed and published; and require paid access, registration, or are freely available to users**			
	Protected and peer reviewed	Only selected users have access, or data set has been published in a peer-reviewed journal	SwissRDL [18], Cancer Registry of Norway [19], and EHRs^a^
	Attributed	User must register to access the data, and the source is referenced	—^b^
	Regulated	User must register to be able to access the data and has no further reference to the generation of the data (eg, scientific publication)	Kaggle [20], Google Dataset Search [21], European Union OpenData.swiss [22], and University of California Irvine Machine Learning Repository [23]
	Free	Access is open source, and data are freely available	—
**Complexity: describes the extent of complexity within a data set (eg, whether the data set contains single, multiple, or longitudinal measurements)**			
	Longitudinal	Univariate or multivariate, measured repeatedly over defined time intervals	Panel study of income dynamics [24]
	Multivariate	Multiple columns or variables (table containing more information than univariate)	—
	Univariate	Only 1 column or variable	Home blood pressure–monitoring pilot: NYU Langone Health EHR [25]
**Sample size: describes the number of samples in the data set**			
	Single	The sample size is 1	Height weight single-variable data [26]
	Small	The sample size is 2 to 100	—
	Medium	The sample size is 101 to 1,000,000	National Health and Nutrition Examination Survey 2013-2014 [27]
	Large	The sample size is >1,000,000	—
**Privacy and liability: describes how well the data set addresses privacy concerns, such as use of encryption, anonymization of participants, and other privacy factors**			
	Encrypted	Data set has been processed through an encryption algorithm and can only be read by authorized parties with the encryption key, and privacy is assured and risk is minimized	Measuring the quality and completeness of medication-related information derived from hospital EHR database (derived data) [28]
	Derived	Data have been analytically preprocessed; there are no privacy and anonymization issues, and liability is minimal; and derived data (not the heart rate of all patients but an average)	—
	Anonymized	Data do not have any identifying particulars or details that would lead to participant identification and have minimal liability	—
	Private	User has private rights or has an established collaboration to access the data (physician with patient data), and liability is reduced because of exclusive rights to the data, but user is responsible for data privacy and safety as determined by law	Twitter data (private) [29]
	Open	Data have no protection measures implemented that protect user identity or privacy; thus, users are responsible for the integrity of the data because there is no information on how they were gathered or managed	—
**Accessibility: describes how the data set can be accessed, such as from a direct download from the web or as a hard copy document**			
	API^c^	Data are accessed through API, and specific data sets are queried and requested by the user and acquired	OpenML (download and API) [30] and OpenData.swiss (API) [22]
	Download	Data are downloadable from the web, but there is minimal functionality (ie, querying is minimal and usually limited in terms of the number of data sets available)	—
	Soft copy	Data are digitally available	USB drive, CD, portable storage, and hard disk
	Hard copy	Data are available only as paper documents	Paper documents
**Periodicity: describes whether the data set is a single snapshot (collected once) or it is designed to be collected and released continuously or periodically**			
	Sequential	Data are measured in a specified periodic or continuous manner	Pervasive computing technologies to continuously assess Alzheimer disease progression and intervention efficacy [31]
	Ad hoc	Data are measured multiple times when necessary (eg, during sickness)	—
	Repeated	Data are generated based on >1 measurement taken at random times	Population census data
	Single	Data are generated based on 1 measurement	
**Standardization: describes whether the data set adheres to, for example, a common international standard or a specific organization**			
	Open metadata	Data are organized according to official standards (eg, Health Level Seven and National Council for Prescription Drug Programs)	A validated smartphone-based assessment of gait and gait variability in Parkinson disease [32]
	Self-metadata	Data are organized using specific descriptors from data providers that describe the structure of the data in detail	—
	Structured	Data are organized in a streamlined and easily interpretable format, but this does not follow any international data set guidelines	Emails, word-processing documents, photographs, and presentations
	None	Data have no clear organization or standardization	—

^a^EHR: electronic health record.

^b^Not available.

^c^API: application programming interface.

**Table 2:** Case study of quality-assessed de novo–generated real-world data^a^.

Variable	Descriptor	Descriptor score	Variable score
Manageability	Protected	100	100 / 1 = 100
Complexity	Longitudinal+ multivariate	100+50	(100 + 50) / 2 = 75
Sample size	Small	33	33 / 1 = 33
Privacy and liability	Anonymized	50	50 / 1 = 50
Accessibility	Application programming interface (API)	100	100 / 1 = 100
Periodicity	Sequential	100	100 / 1 = 100
Standardization	Self-metadata	66	66 / 1 = 66

^a^Cumulative score = 100 + 75 + 33 + 50 + 100 + 100 + 66 = 524; quality identifier = (524 / 7) / 100 × 5 = 3.74.

The RWD-Cockpit system is based on a database that manages the available data sets as well as the scoring of data sets. Data sets are assigned descriptors using database object relations. When querying data sets, related flags are automatically fetched for each data set; the data set score is calculated simultaneously and then displayed to users. This simultaneous scoring mechanism allows for assessments to change dynamically over time if there are changes to the variables or the scores. In addition, personal data sets and data sets not provided by the application can also be self-scored through the *Help* link in the application. A user can click on *Enable score calculator* and easily choose the appropriate descriptors for a data set, after which a score is generated automatically.

Furthermore, a statistics page was designed to provide an internet-based chart of the data sets within the application to track global trends in RWD quality assessments from several sources (eg, Kaggle). The global average quality identifier scores of all available data sets and for each type—molecular, phenotypical, and social—are displayed. In terms of technology, the RWD-Cockpit system is developed as an Angular web application on top of a Loopback server, and the data are stored in a MongoDB database. This architecture is robust and straightforward to deploy and maintain. The RWD-Cockpit web application is free to use, and users are encouraged to input their data set descriptions and add their data sets in the database. For data sets associated with a publication, the publication reference is cited.

### De Novo–Generated RWD as a Case Study

The measurements were performed with a ribbon containing electrodes that is placed underneath a fitted sheet in a bed at chest height. The electrodes quantitatively measure humidity, which is correlated to sweat, during sleep (Multimedia Appendix 1). In addition, the sensor measures the temperature at the ribbon and in the room using a printed circuit board (Multimedia Appendix 2 and Multimedia Appendix 3). Resistance and temperature measurements are taken throughout the night by 2 electrodes within the ribbon, and the values collected are transferred to a moisture sensor connected to a printed circuit board (Multimedia Appendix 4).

## Results

### A Straightforward Web Tool That Assesses RWD Data Sets

Many challenges [33] and information parameters to guide RWD appropriateness for research questions have been proposed [34]. However, a framework that translates information (eg, quantifying parameters such as the importance of longitudinal data, accessibility, and publication as a metric that indicates quality [34]) into an evaluation framework is still missing. We created the RWD-Cockpit [35], a straightforward web tool that assesses RWD data sets using standard and customizable variables (Figures 2 and 3). This web application provides a platform to search for, and view, quality-scored RWD data sets. Furthermore, it provides a flexible benchmarking data quality–scoring tool for new user-acquired RWD data sets. Users can search for RWD data sets that fulfill user-selected quality variables and score criteria, as well as set a standard that other users within the same institution or across institutions or regulatory agencies may use. Currently, 106 quality-scored RWD data sets from a variety of sources and areas are available through the RWD-Cockpit, and more are continually added. As new guidelines and laws are passed concerning the use of RWD, novel variables and descriptors can be added as needed by the administrators.

In the RWD-Cockpit, RWD data sets were scored based on 7 variables that were identified to be important metrics in determining data quality to address the challenges in adopting RWD for regulatory decision-making [11,36]. The proposed variables are *manageability*, *complexity, sample size, privacy and liability, accessibility, periodicity,* and *standardization,* and they are described in detail in Table 1. Each variable contains 3 to 5 *descriptors* that describe specific characteristics that apply to a data set, such as *multivariate* or *longitudinal* measurements (Table 1). Each variable has been identified based on its impact on the overall usability of data. Because of the broad landscape of potential use cases for RWD, the identified variables do not consider case-specific suitability or content but create a generalized framework to assess RWD. *Manageability* is an important variable necessary because of the broad diversity and almost nonexistent limitations on what data identify as RWD (Table 1). The level of data management [36] can be related to the general quality and trustworthiness of the data. A higher score is proposed for either peer-reviewed data sets or data that require additional efforts with regard to data management. The *complexity* of data extends the use-case coverage of the data sets. Univariate data might offer a base to solve single research questions but lack the depth of potential insights. A proposed option to achieve an increased score for *complexity* is to provide or generate diverse data, enabling the data to be integrated into a broader field of use cases. The variable *sample size* is of great importance because RWD are intended to show real-world insights. Real-world behavior can be reflected better in data from large numbers of individuals compared with information on a single individual. The *sample size* of a data set can be increased at any time, given that the circumstances of the data acquisition, such as used devices, remain the same for each data point. The level of compliance with given data privacy regulations, as represented in the variable *privacy and liability*, can provide further insights on data quality and trustworthiness. Open RWD without data protection measures have a high potential of being simulated data, whereas reliable data sources are compelled to comply with given regulations. The application of data anonymization or encryption measures and compliance with European Union standards (General Data Protection Regulation) or US standards (Health Insurance Portability and Accountability Act) results in a higher assigned score. The state in which data are being transferred and stored takes on a relevant role when intending to extract RWE from RWD; thus, the variable *accessibility* has been identified as a relevant factor. Hard copy of data might provide a wide range of content but increases efforts in preprocessing and requires the transformation from analog to digital. The most efficient method to implement the accessibility variable is to provide the data through an application programming interface. Using an application programming interface allows users to specifically query the data of interest and, furthermore, provides direct digital access to the data. Similar to *complexity* and *sample size*, *periodicity* has been identified as a relevant parameter because of the depth of information. Single snapshots of individuals reflect acute states, whereas data acquired at different points in time of the same individual can generate deeper insights, which indicates reproducibility of the method. Collecting data from individuals repeatedly according to a defined time plan leads to a 2-fold benefit: first, the ability to create an average overview on individuals, and second, the ability to identify time-related patterns or progression. The last variable *standardization* is required to further increase trustworthiness and practicality. Applying state-of-the-art health care data standards to RWD generates a more direct path to use the data, whereas unstructured data or data that follow nonconventional standards require increased efforts with regard to their understanding and use. To achieve a higher score in *standardization*, it is proposed to identify potential community standards or frameworks and apply these to the data set.

The overall average score of all data sets in the RWD-Cockpit application was 2.80, with social data sets scoring 2.90, molecular data sets scoring 2.86, and phenotypical data sets scoring 2.83.

### Case Studies

To provide a practical example on the benefits of using the RWD-Cockpit, quality metrics were calculated in two case studies: (1) a practical case study using temperature data during sleep and (2) two of the publicly available data sets in the database.

To evaluate the applicability and usability of the RWD-Cockpit web application on de novo–generated data, a case study was performed to generate temperature RWD during sleep [37]. The RWD generators were asked to use the RWD-Cockpit application on their data sets, determine the value the application provided, and use it to find quality-scored data sets that were useful for their company. Their preliminary data sets scored 3.74 out of 5, which is determined to be *good* within the application (Table 2). On the basis of this score, the data generators identified variables that can be improved upon within their data set, such as increasing the sample size, publishing the data in a peer-reviewed journal, encrypting the data, and organizing the data according to officially recognized standards, such as Health Level Seven. These developments will increase the quality of their data set, making it more likely to be adopted in a health care environment as a novel digital biomarker for health and to be accepted as a health tool. Furthermore, the data generators identified within the RWD-Cockpit application several scored RWD data sets related to sleep and health that were meaningful and independently collected to further develop their product. This case study validated the purpose and value of the RWD-Cockpit web application for use.

We selected and scored 2 different health care–related data sets to demonstrate how their respective scores reflect the low and high quality of the RWD Table 2 and Table 3. The first data set, named *Height Weight Single Variable data*, studies the relationship between the height and weight of a person, predicting the probabilistic weight for a given height from a list of heights and weights [26]. The second RWD data set, *National Health and Nutrition Examination Survey 2013-2014*, consists of health measurements and surveys (eg, demographics and laboratory measurements) of approximately 5000 individuals across the country over a 2-year period conducted by officials at the US Centers for Disease Control and Prevention [27]. These data include measurements conducted by physicians and laboratories, as well as self-reported measurements. When quality identifiers are compared between the 2 data sets, the second data set scores higher at 3.67 versus the score of 1.47 of the first data set Table 3. The major differences between the 2 data sets are primarily visualized in 5 of the 7 variables: *manageability, complexity*, *sample size*, *periodicity*, and s*tandardization*. Whereas the first data set contains only 35 different samples, the second data set has collected data from 5000 different samples, resulting in a higher score. Regarding *complexity*, the first data set is *univariate*, whereas the second data set is both *longitudinal* and *multivariate*, thus scoring higher than the first data set. Additional differences in descriptors and scores are detailed in Table 1.

The score of the Height Weight Single Variable data set can be improved on various aspects. The data set can be extended with additional individuals to increase the *sample size* variable from *small* to *medium*, and further information related to these individuals can also be used as an extension of the data set. Furthermore, a structured plan can be applied to measure the required information for the data set at specific time points, further increasing the *periodicity* score of the data set. The *periodicity* variable provides the ability to data providers to define the time ranges and number of independent measurements without constraints, allowing measurements on the same day to be graded *repeatedly* or even ad hoc. In addition, when repeated measurements of the same individuals are performed multiple times, the *complexity* score is affected as well, moving from *univariate* to *longitudinal*. On the basis of the additions to the data set, an overall data dictionary can be created to improve the *standardization* score from the current *structured* to *self-metadata*. The extended data set can further be used to conduct research on, granting the option to receive peer reviews on the data set or to move the *manageability* score from *free* to either *attributed* or *peer reviewed*. With regard to *privacy and liability* and *accessibility*, *anonymized* and *download* already provide high scores while covering the variables appropriately for this kind of data set; thus, further improvements are not necessarily required.

The described adjustments and additions to the data set can cause a significant impact on the overall score of the data set. The Height Weight Single Variable data set can increase its score from 1.47, considered a *poor* data set by the platform, to 3.60, reaching the classification *good*. Besides raising the score, the proposed adjustments strongly improve the usability and reliability of the data set. The data set can cover a much wider range of use cases and gain trust of stakeholders. A complete overview of potential improvements to the data set is shown in Table 3.

This simple scoring of RWD enables investigators and health care stakeholders to get a general overview of the suitability of the data sets in relation to the decisions the data will affect. It also allows users to apply the same standards for assessing RWD quality. For high-impact health-related decisions, RWD should be of high quality and scrutinized for validity. The ability to add new potential descriptors to any variable or add completely new variables makes the RWD-Cockpit dynamic and adaptable. In addition, new governmental regulations can be implemented easily by adding new variables. Scoring can be adjusted according to the regulations for each country or specific regulatory or industry-specific requirements. By using the RWD-Cockpit, a fast evaluation of RWD quality can be achieved and the data sets can be scored depending on the fulfilled requirements for further consideration.

**Table 3:** Potential improvements to a case study of publicly available real-world data^a^.

Variable	Value	Score	Improvement	Value after improvement	Score after improvement
Manageability	Free	0	Move from providing the data freely to attributed access or publish the data in a peer-reviewed journal	Attributed	66
Complexity	Univariate	0	Extend the data set by collecting more information on participants at different time points	Longitudinal	100
Sample size	Small	33	Increase the number of individuals included in the data set to >100 participants	Medium	66
Privacy and liability	Anonymized	75	Privacy and liability are already handled appropriately	Anonymized	75
Accessibility	Download	66	Access through download is appropriate for data such as these	Download	66
Periodicity	Single	0	Develop a plan for when the participants will be examined again and extend the data set in defined time steps	Ad hoc	66
Standardization	Structured	33	Create a data dictionary clearly stating the structure of the data set	Self-metadata	66

^a^Score before changes: 1.47; score after changes: 3.60.

## Discussion

### Setting Standards for RWD: Opportunities and Challenges

Digital technological advancements such as measurement of digital biomarkers [13], wide implementation and use of electronic health records, and social media have generated a wealth of health-related data that can potentially be leveraged to generate valuable RWE. Biopharmaceutical companies have already begun to harvest the plethora of data and to integrate the data for new drug applications and postmarket analysis of various therapeutics [4]. Integration of RWD is valuable and has the potential to reduce the huge health care expenditure costs without lowering the standards for evidence [38]. Regulatory authorities such as the FDA or the EMA face challenges when it comes to consideration of RWE generated from RWD in regulatory decision-making and drug approval [2,11]. Some of the challenges [33] the agencies must overcome are related to ensuring the quality of data and providing frameworks for consideration. Without guiding regulations, the currently expanding use of RWD in studies [26,39] fails to follow industry standards. In addition, a robust standard for data sets must be implemented following the Findability, Accessibility, Interoperability, and Reusability (FAIR) principles [40]. However, these principles are related to data upstream the RWD quality assessment, and there are no guidelines from federal agencies on how to standardize data sets or individual data points. Several attempts are being made by groups such as the Observational Medical Outcomes Partnership, the Sentinel System, and the National Patient-Centered Clinical Research Network to partially address standardization by using systems such as the common data models that standardize terminology and transform databases into a similar format and representation [41]. Another strategy is to evaluate apps that generate the RWD [42,43]; however, this would translate into a discrepancy in the quality evaluation process that would reflect on, and differ in, the steps specific to the application areas of disease and usability. In contrast, information regarding RWD quality in the community is mainly reported in the form of characterization [34], which lacks the formality of a metric.

The complexity of RWD could be greatly reduced by providing a standard for the industry and other health care stakeholders. For RWD to be accepted into mainstay biopharmaceutical pipelines, regulatory agencies must first begin defining and setting standards on what data can be considered valid to be used for health-related decisions. Up to this point, no tools have been provided for the quality assessment of RWD. In addition, generation of RWD is still relatively siloed, with industry-sponsored studies being the main contributors [39]. To harness the creative power of the broader community (eg, academic centers that do not have the resources to generate such data themselves), the process of identifying and assessing the quality of RWD must be streamlined [13]. Nevertheless, considering RWD as a source of evidence in clinical or regulatory decision-making is a process under development. Different variables for the consideration of RWD in making health-related decisions are important and need to be identified appropriately for a variety of end-use analyses. In addition, participants and patients involved in studies that generate RWD must provide complete and trustworthy information. When collecting RWD data sets outside of RCTs, assurance must be given on, for example, data reliability, integrity, availability, and, not least, completeness. The RWD-Cockpit provides an easy-to-use and traceable first general assessment of RWD quality, which is applicable to a wide variety of data sets. Current regulations do not provide a sufficient framework for inclusion of RWD in studies investigating diseases other than orphan diseases or oncological diseases [44-46]. However, the value and possibilities when considering RWD during the whole product lifecycle instead of only postmarket authorization are recognized across disease areas. The RWD-Cockpit enables health care stakeholders to obtain preliminary quality information across RWD data sets. Users can use this preliminary criterion when searching for, and selecting, specific data sets for consideration in investigations. The web-based RWD-Cockpit application might provide an initial standard blueprint for regulatory authorities considering preliminary approval of the use of RWD in different settings.

The RWD-Cockpit allows users to score data sets independently, where they can assess the scores according to the RWD formula and further choose whether they wish to publish these data sets in the RWD-Cockpit. In addition, the results of the case studies demonstrate the applicability and usability of the RWD-Cockpit application to the wider community. Future versions of the RWD-Cockpit or frameworks for the assessment of RWD quality before further selection and analyses might also consider additional information on the data-generation process, including variables focusing on used devices or firmware. Furthermore, information can be included on whether the data have been centralized by a single institution or person or distributed and combined from multiple sources. Another potential improvement to the RWD-Cockpit could result from the automation of the scoring process. An automated machine-performed grading mechanism could potentially lead to an increase in consistency compared with the current manual grading by an individual. Another potential future useful feature could enable users to score their own RWD data sets based on their own criteria and similarly apply these quality criteria across a single institution or through multiple institutions.

### Conclusions

The RWD-Cockpit web application is designed to enable a fast and reliable scoring system for evaluating the multi-metric quality of RWD data sets. It aims to reduce preliminary issues related to quality assessment of RWD and streamline the discovery of valuable RWD data sets, and it has the potential to be used in clinical settings. The application of this tool in the context of RWD is diverse and expandable, as demonstrated through the case studies. With the advent of digital medicine and the increasing challenges in data and metadata standards of RWD, there is a pressing need to develop frameworks and tools that represent RWD quality in a metric, comprehensible, and traceable manner and can serve as a standard across data sources and disease areas. The RWD-Cockpit represents a first metric proposal in this direction; however, further community efforts are urgently needed.

## Appendix

Multimedia Appendix 1 The Q-Strip is connected to a printed circuit board where the resistance between 2 electrodes is measured. The data collected are sent to a secure database.

Multimedia Appendix 2 The Q-Strip is placed at chest height in a bed to measure nocturnal sweat best accurately.

Multimedia Appendix 3 The longitudinal data are collected and then visualized in the Q-Strip software. The x-axis represents date and time and the y-axis represents the resistance value corresponding to amount of sweat detected.

Multimedia Appendix 4 Example of quality-assessed real-world data.

Multimedia Appendix 5 Q-strip product is a ribbon that contains electrodes which measure humidity. The resistance between the 2 electrodes within the strip measure humidity which is then correlated to sweat.

## Abbreviations

EMA: European Medicines Agency
FDA: Food and Drug Administration
RCT: randomized controlled trial
RWD: real-world data
RWE: real-world evidence
