# Supplementary figures and images for: RWD-Cockpit: Application for Quality Assessment of Real-world Data
Source: JMIR Form Res. 2022 Oct 18;6(10):e29920. doi: 10.2196/29920 (PMC9627468; doi:10.2196/29920)

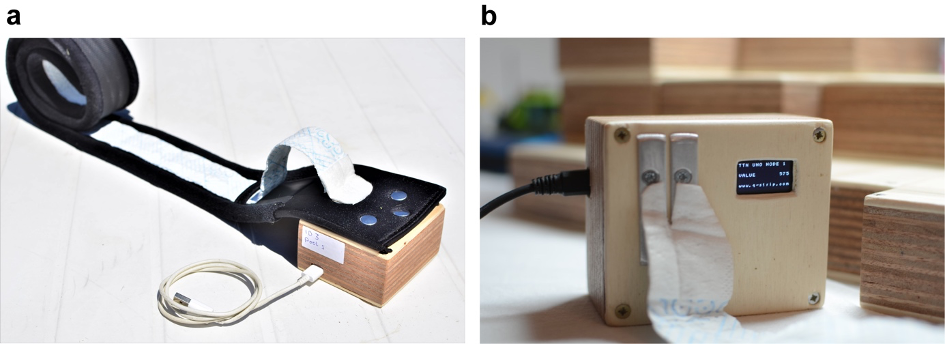

Supplement: Multimedia Appendix 1 [file formative_v6i10e29920_app1.png]

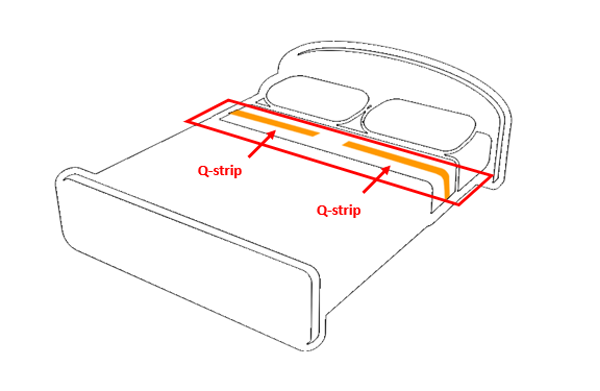

Supplement: Multimedia Appendix 2 [file formative_v6i10e29920_app2.png]

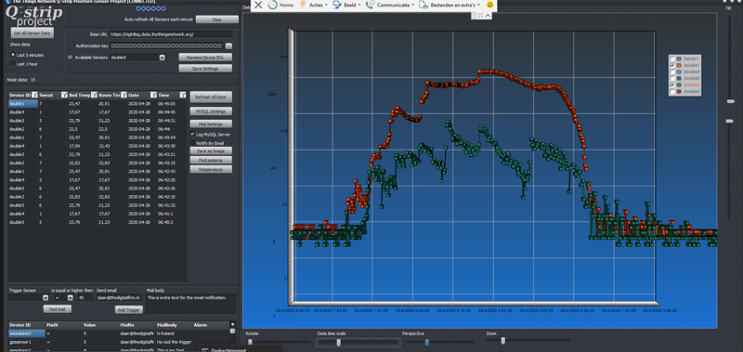

Supplement: Multimedia Appendix 3 [file formative_v6i10e29920_app3.png]

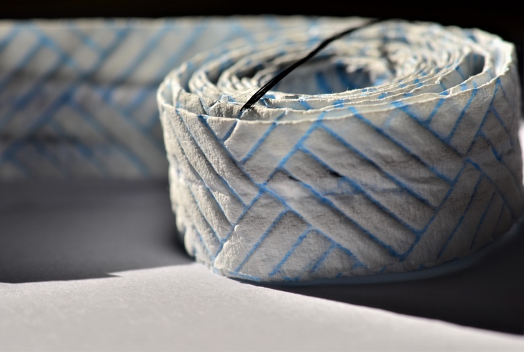

Supplement: Multimedia Appendix 5 [file formative_v6i10e29920_app5.png]
