# Supplementary material for: RWD-Cockpit: Application for Quality Assessment of Real-world Data
Source: JMIR Form Res. 2022 Oct 18;6(10):e29920. doi: 10.2196/29920 (PMC9627468; doi:10.2196/29920)
Supplement: Multimedia Appendix 4 [file formative_v6i10e29920_app4.pdf]

Multimedia Appendix 5. Example of quality-assessed RWD

	Dataset name					
	Height weight single variable data (Kaggle)			*National Health and Nutrition Examination Survey 2013-2014 (CDC)*		
**Variable**	**Descriptor**	**Desc. Score**	**Var. score**	**Descriptor**	**Desc score**	**Var. score**
Manageability	Free	0	0 / 1 = 0	Attributed	66	66 / 1 = 66
Complexity	Univariate	0	0 / 1 = 0	Longitudinal<br>Multivariate	100<br>50	(100+50)/2 =75
Sample size	Small	33	33 / 1 = 33	Medium	66	66 / 1 = 66
Privacy & Liability	Anonymized	50	50 / 1 = 50	Anonymized	50	50 / 1 = 50
Accessibility	Download	66	66 / 1 = 66	Download	66	66 / 1 = 66
Periodicity	Single	0	0 / 1 = 0	Sequential	100	100 / 1 = 100
Standardization	Structured	33	33 / 1 = 33	Self-Metadata	66	66 / 1 = 66
**Cumulative score**	0 + 0 + 33 + 50 + 66 + 0 + 33 = 182			66 + 75+ 66+ 50 + 66 + 100 + 66 = 489		
**Quality Identifier**	(182 / 7) / 100 * 5 = **1.30**			(489 / 7) / 100 * 5 = **3.49**		

(Desc. = Descriptor, Var. = Variable)

RWD-Cockpit function descriptions (http://rwd.aihealth.ch/)

1) **Help**
   - Helps the user understand each descriptor and how the score is affected.
   - Enables score calculator.
   - The RWD-Cockpit cannot host (Manageability) protected data, this is why we host the metadata (e.g., similar an index).

2) **Browse**: is a google-like, minimal interface.
   - Search for keyword that can appear anywhere in the metadata such as title, or description for all available fields as well as the name of institution that is making it available.
   - If one of the tags is selected, the results represent an OR combination of this tag. If more tags are selected, more results are returned.
   - Results return the metadata and link to the original RWD dataset. A list of each dataset found and its corresponding metadata is shown. Under

the title, a link is provided that brings the user to the dataset. On the right, the score according to our rating system is reported. The highest scored dataset, with the better quality, is on top, and search results return data in descending order, from best to lowest score.

- Search for datasets to include in the RWD-Cockpit was performed manually. In the future, crowdsourcing options are desirable so that more community contributors can score and insert dataset references.
- An advanced search can be performed for a certain keyword, but dataset must contain one of those tags. Each tag describes a characteristic about the data and corresponds to a partial value of the score. Search is enabled also by each of the values of the variables.
- Tags describe all properties of the dataset for each variable.
- Hovering on top of each tag, a description appears. Tags represent descriptors, possible values for variables.

3) **Statistics**: is an overview of all the datasets indexed in the RWD-Cockpit database according to their variables and descriptors.
    - The information is arranged by quality (average score of the dataset that belong to that group) and quantity (the number of datasets).
    - If a certain dataset has a score above the average score, that is better than the average dataset in the group.
    - As the user clicks in each category, a subcategory is shown: Dataset Type Social/Molecular/Phenotypic will lead to Manageability, Complexity, Privacy, Accessibility, Periodicity, Standardization, Sample size. For example, Molecular has 13 datasets and all have complete fields (it is optional to have values for all variables, however in this case we have data for all variables and no missing values NA). In the case of a lower-quality dataset, the single numbers would be less because of missing data.
    - There is an average descriptor quality for each variable. For example, Phenotypical has 91 datasets and the average quality for the dataset for that variable is shown (e.g., *Manageability* is 43%). If the user clicks on Manageability, the descriptors are shown with different possible values visualized. All datasets here have either Free, Attributed, or

Protected and/or peer-reviewed. Thus, the average score of the datasets grouped by the descriptor of the variable can be visualized. If the user selects the free datasets, the source is visualized. We can change the selection at the navigator bar right above the chart, with the same version of the same operations: e.g., change from “Free” to “Attributed” selected datasets. The drop-down menu options can reproduce this behavior. The menu is user-friendly with screen reader application to improve accessibility of the RWD-Cockpit.
